# Difference in Occlusal Contacts Obtained with Conventional Orthodontic and Clear Aligner Therapy: A Pilot Study

**DOI:** 10.3390/dj14030169

**Published:** 2026-03-13

**Authors:** Giorgio Oliva, Roberta Maddaluno, Roberto Rongo, Gerarda Buonocore, Rosa Valletta, Ambrosina Michelotti, Vincenzo D’Antò

**Affiliations:** 1 Section of Orthodontics, Department of Neuroscience, Reproductive Sciences and Oral Sciences, University of Naples Federico II, Sergio Pansini Street, 5/Edificio 14, 80131 Naples, Italy; giorgio.oliva@unina.it (G.O.); roberto.rongo@unina.it (R.R.);; 2 Independent Researcher, 80131 Naples, Italy

**Keywords:** occlusal contacts, aligners, fixed appliance

## Abstract

**Background/Objectives**: The achievement of stable and functional occlusal contacts represents a key objective of orthodontic treatment, particularly in growing patients. Evidence comparing the effectiveness of these two modalities in establishing adequate occlusal contacts in growing patients remains limited. This study aimed to evaluate and compare occlusal contact characteristics following clear aligner therapy (CAT) and fixed orthodontic therapy (FAT). **Methods**: Twenty-four growing patients (<18 years with permanent dentition) were included in the study and divided into two groups: 12 patients treated with fixed appliances and 12 treated with clear aligners. Post-treatment digital dental scans were analyzed to assess occlusal contacts. Contacts were calculated as the minimum distance between upper and lower arches using a color-map analysis. The following outcomes were evaluated: Maximum Contact Point (MCP), occlusal contact surface (OCS, ≤50 μm from MCP), near occlusal contact surface (NOCS, ≤350 μm), half mm (≤0.5 mm), and one mm (≤1 mm). Total occlusal contacts, antero-posterior distribution, left–right asymmetry, and single-tooth contacts were assessed. **Results**: The FAT group showed higher total occlusal contact values in OCS compared to the CAT group (*p* < 0.05). Statistical difference was also observed in the antero-posterior ratio, with FAT presenting fewer anterior contacts in OCS, NOCS, half-mm, and one-mm measurements (*p* < 0.05). No significant differences were found between groups in terms of left–right asymmetry or post-treatment single-tooth contacts, except for the second premolar, which exhibited higher contacts in the FAT group (*p* < 0.05). **Conclusions**: Fixed orthodontic treatment is more effective than aligners in achieving adequate occlusal contacts, with differences limited to tight contacts and antero-posterior occlusal distribution.

## 1. Introduction

The use of orthodontic aligners (CAT) as a more aesthetic and comfortable therapeutic alternative to fixed traditional orthodontic therapy (FAT) has increased over the past few years [1]. Dental malocclusion is highly prevalent among children and adolescents worldwide [2] and can negatively affect patients’ psychological and social well-being [3]. Consequently, effective orthodontic treatment plays a crucial role not only in improving occlusion but also in enhancing overall quality of life. However, the advantages of CAT extend beyond aesthetics. CAT has been found to improve oral hygiene, periodontal health, soft tissue irritation and patients’ comfort [4,5,6]. Furthermore, the development of three-dimensional (3D) digital technologies and dental materials has led to a considerable increase in CAT utilization among clinicians [7,8]. A recent systematic review by Alhafi et al. found no significant differences in treatment quality between CAT and FAT in non-extraction cases with mild malocclusions [9]. However, the quality of occlusal contacts achieved with the two therapies seems to differ, especially in more complex cases [9,10]. A balanced distribution of occlusal contacts is widely regarded as essential for the long-term stability of treatment outcomes [11,12,13].

Several methods have been proposed to analyze occlusal contacts. Kassas et al. [14] evaluated pre- and post-treatment Invisalign records using the American Board of Orthodontics (ABO) Model Grading System (MGS). In most published studies, occlusal contacts have been assessed using either the ABO grading system [15] or the Peer Assessment Rating (PAR) index [16,17,18,19,20]. A difference in ABO grading score, especially in the occlusal contact, can be found in more complex cases.

However, both the ABO and PAR scoring systems rely on manual assessments and provide limited information regarding the spatial distribution of occlusal contacts.

A different approach is the use of T-scan to evaluate occlusal contact. Using this method, Cohen-Lévy et al. [21] found no significant differences in occlusal contact quality between CAT and FAT. Interestingly, these authors have investigated the symmetry of occlusal contact obtained with the treatments. Both therapies produce slightly asymmetric occlusal contacts.

A third approach relies on a distance color map created with intraoral scans [22,23,24]. In particular, Frenkel et al. [23] did not find any difference between FAT and CAT using this method. These authors reported that occlusal contact changes were greater in older patients, suggesting that age may influence treatment outcomes. Even if these authors have analyzed antero-posterior differences in occlusal contacts, they have not analyzed left–right asymmetries. The primary aim of this pilot study is to evaluate the minimum occlusal distance on each dental element before and after CAT and FAT. The secondary aim is to assess total occlusal contacts before and after treatment with clear aligners and fixed appliances, as well as to evaluate the anterior–posterior ratio and potential asymmetries between the right and left hemiarches.

## 2. Materials and Methods

This is a retrospective cohort study. Twenty-four patients were selected from a database belonging to the department of Orthodontic AOU “Federico II”: 12 patients were treated with FAT, while 12 with CAT. The Ethics Committee of the University of Naples Federico II (Italy) approved the research protocols with the following codes: 179 2023. Consent for personal data processing for scientific purposes was given by each patient. The inclusion criteria were: age less than 18 years old with permanent dentition, patients with periodontal health, patients who have completed CAT or FAT, patients with complete radiographic records (OPT and latero-lateral X-ray) at the beginning of the therapy, patients with pre-treatment intraoral scans, and patients with post-treatment intraoral scans (within one week from the end of therapy). The exclusion criteria were: patients presenting cognitive/behavioral conditions that prevented compliance with orthodontic procedures as judged by the clinicians; patients undergoing orthognathic surgery; patients requiring extractive treatment; patients with congenital absence of teeth (excluding third molars), including unilateral or bilateral agenesis, when space closure or prosthetic compensation could influence final occlusal outcomes; patients diagnosed with dental ankylosis, identified clinically and/or radiographically at any time prior to the end of active treatment; patients with severe skeletal discrepancies; patients with temporomandibular disorders; presence of retention appliances during scanning; additional appliance usage.

All FAT cases were treated using standard MBT straight-wire technique with the standardized archwire sequence: 0.13 NiTi, 0.16 NiTi, 0.19 × 0.25 NiTi and 0.19 × 0.25 SS posted. Finishing procedures were completed with 0.16 SS archwire. Brackets were bonded from the left second premolar to the right second premolar, while buccal tubes were used on the molars in order to minimize the potential occlusal alteration by the placement of bands. According to individual sagittal correction needs, Class II/III and/or vertical settling elastics were worn full-time during the working phase, except at meals. Entry into the finishing stage was permitted only after adequate arch coordination had been achieved and proper intercuspation was clinically verified.

All CAT cases were treated using Invisalign clear aligners (Align Technology, San Jose, CA, USA). The digital treatment plan (Clincheck^®^pro version 6) defined the intended occlusal objectives and served as a reference for clinical monitoring during orthodontic treatment. Composite attachments were used when required to enhance aligner retention and optimize the predictability of tooth movement. Once adequate alignment and leveling had been achieved, intermaxillary elastics were prescribed, if necessary, for sagittal correction, using precision cuts or other bonded auxiliaries. Elastics were maintained as required to accomplish the planned sagittal correction. At the end of the initial set of aligners, the achieved occlusion was clinically compared with the planned objectives. If arch coordination, alignment or intercuspation were considered inadequate, additional aligners were requested to improve the occlusal outcome.

Occlusal contact analysis was performed using anonymized file identification codes in order to ensure operator blinding to participant identity and allocation. Intra-operator reliability was evaluated by repeating the measurements in a randomly selected subset of patients (10 patients from the total sample). The same operator repeated the analysis after a washout interval of one month to minimize memory bias.

Inter-operator reliability was assessed on the same subset of records. A second independent and comparably trained operator, blinded to the first examiner’s results, repeated the analytical procedures as per the standardized protocol. Intra- and inter-operator reliability showed excellent consistency, with intraclass correlation coefficients exceeding 0.95 in both assessments, confirming the reproducibility of the measurement procedure.

For each patient, occlusal contacts were calculated as the minimum distance between the upper and lower scans. The minimum distance was calculated on each tooth through the creation of a color map and the identification of the least distant points. On the front teeth, a single distance was detected for each tooth. On the posterior teeth (premolars and molars), two distances were calculated for each tooth, buccal and palatal. The procedure used has already been validated in the literature [22]. Different outcomes were used for the evaluation of occlusal contacts: “Maximum Contact Point” defined as the point with the minimum interocclusal distance on each tooth’s occlusal surface; “occlusal contact surface” (OCS) defined as the area comprising all points within 50 μm of the opposing arch; “near occlusal contact surface” (NOCS) defined as the area comprising all points within 350 mic μm of the opposing arch; “half mm” defined as the area comprising all points within 0.5 mm of the opposing arch; “one mm” defined as the area comprising all points within 1 mm of the opposing arch.

**Figure 1:**
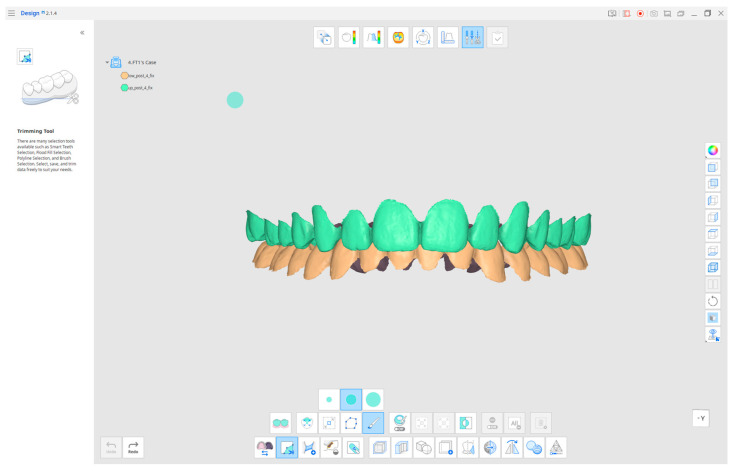
The image shows a preliminary step. The teeth are the only part of the model selected and used for the analysis.

To obtain these measures, the data have been analyzed as follows. Upper and lower three-dimensional scans were imported into Medit Link (Version 3.3.0; Medit-Seoul, Republic of Korea). The Medit Design App (Version 2.1.4) enabled users to select individual teeth from intraoral scans through its “Edit Mode” and “Smart teeth selection” features, which produced STL files for export (Figure 1).

**Figure 2:**
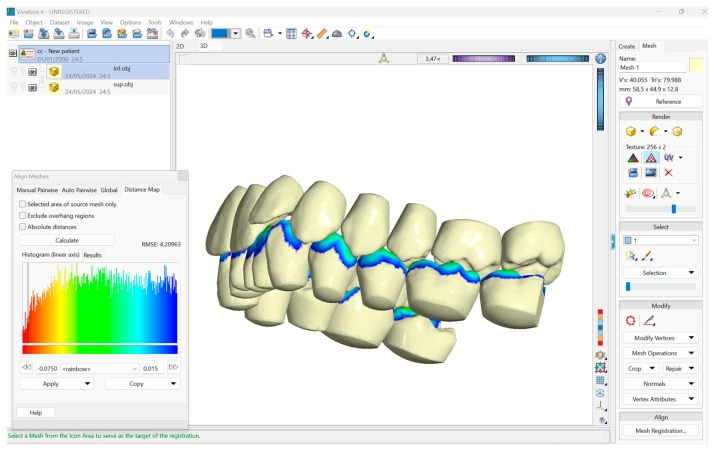
The image shows one step of the analysis performed. The two models imported were analyzed using two distance color maps. The panel on the left lets the user choose the distances for the calculation. In the figure commas are used for decimal separation.

STL files were imported into Viewbox 4 (Version 4.1.2.1, dHAL Software, Kifissia, Greece). The “Mesh Registration” tool produced a distance color map, which showed the lower arch (source) against the upper arch (target) without any exclusion of overhanging areas. The Maximum Contact Point for each tooth was determined through the export of vertex distances. The researchers used this value to establish four distance thresholds, which included OCS (Maximum Contact Point + 50 μm), NOCS (+350 μm), half mm (+0.5 mm), and one mm (+1 mm). The program calculated total occlusal contact surfaces for each threshold value, which appeared on the distance map (Figure 2).

The analysis of asymmetries needed the lower arch to receive quadrant division through the “Selected area of the source mesh only” option. The anterior–posterior ratio was calculated as anterior (central incisor, lateral incisor, canine)/posterior (first premolar, second premolar, first molar, second molar). Left-right asymmetry was calculated as 1 − (right contacts/left contacts).

The required sample size was based on the results of Cohen-Lévy et al. [21]. These authors measured anterior–posterior contact surface ratios in patients who received clear aligner or fixed appliance treatment and found their standard deviation values between 0.09 and 0.11. Julious [25] recommends that pilot studies should include 12 participants per group to measure variability effectively, which will help researchers design their upcoming big clinical trials. The research needs 12 participants per group to identify a 0.10 difference in the anterior–posterior ratio with 80% power at α = 0.05 (two-sided test). The measured difference amounts to 40% of the established baseline ratio value (0.22–0.28) (Cohen-Lévy et al. [21]). The research design of this pilot study aimed to determine effect size values and the measurement range for upcoming validation studies.

The researchers evaluated single-tooth occlusal contacts for both CAT and FAT before treatment and after treatment by studying each contralateral tooth pair as a single unit. With our sample, some patients in T0 did not have the second molar, which was not considered in the statistical analysis of occlusal contacts. All variables had a non-normal distribution and were described as Median ± IQR. Two different tests were used to analyze occlusal contacts at T0 and T1 in both groups. Given the pilot nature of the study, the primary aim of the statistical analysis was to compare the two treatment modalities at predefined observation times, with particular emphasis on post-treatment differences (T1). This approach was chosen to obtain preliminary estimates of variability and effect size for the design of future adequately powered longitudinal studies. The non-parametric Fligner–Killeen test measured occlusal contacts’ homogeneity among the two groups before and after treatment. The unpaired two-sample Wilcoxon test compared occlusal contacts between the two samples at T0 and T1. All conducted tests were two-sided, and *p*-value < 0.05 *, *p*-value < 0.005 **, or *p*-value < 0.001 * were considered statistically significant. R (version 4.4.1, Lucent Technologies, Murray Hill, NJ, USA) was used for the statistical analysis.

## 3. Results

Twelve patients treated with FAT and 12 treated with CAT were recruited for the study. Table 1 shows the characteristics of the two samples at T0.

### 3.1. Single Tooth-Maximum Contact Point

At baseline, both FAT and CAT reported, for each tooth, lower occlusal contacts compared to post-treatment evaluation (Table 2).

At T1, no statistically significant differences in occlusal contacts were found between groups. Only for the second premolar, a statistical discrepancy was described with lower occlusal contacts in the CAT group compared to the FAT group (Table 3). Less variability in post-treatment single-tooth occlusal contacts was seen in FAT in comparison with CAT, and this difference between groups was statistically significant for the lateral incisor and canine (*p*-value < 0.05 *), second premolar (*p*-value < 0.01 **) and first molar (*p*-value < 0.001 ***).

### 3.2. Total Occlusal Contacts

When evaluating total occlusal contacts, at T1, there was a statistical difference between FAT and CAT groups in occlusal contact surface (OCS), with higher values reported in FAT (*p*-value < 0.05 *). No differences were found evaluating NOCS, half mm and one mm between groups (Table 4). Based on the OCS data from this pilot study (CAT: median 0.43 mm^2^, IQR 0.84; FAT: median 1.27 mm^2^, IQR 1.38), a future confirmatory trial using non-parametric tests aiming to detect the observed difference of 0.84 mm^2^ with 80% power at α = 0.05 would require approximately 26 patients per group.

As shown in Table 5, the discrepancy between occlusal contact surface variability was statistically significant, with more variable values in FAT compared to CAT (*p*-value < 0.05 *).

### 3.3. Antero-Posterior Ratio

At T1, CAT showed major anterior occlusal contacts in all the outcomes considered (*p*-value < 0.05) (Table 6) compared to FAT, particularly in the OCS evaluation, where no contacts were described in the second group. Moreover, FAT reported significantly less variability in antero-posterior ratio values than CAT when considering OCS (*p*-value < 0.001 ***), NOCS (*p*-value < 0.01 **), half mm and one mm (*p*-value < 0.05 *).

### 3.4. Asymmetries Between Left and Right Hemiarches

CAT and FAT reported comparable values of asymmetry between left and right hemiarches (Table 7). Similarly, asymmetry variability was not different between groups.

## 4. Discussion

The primary aim of this pilot study was to evaluate the occlusal distance before and after CAT and FAT, with post-treatment records obtained immediately after active therapy in order to capture the occlusal relationships achieved after the orthodontic treatment, prior to neuromuscular adaptation and spontaneous settling. We found an increase in occlusal contacts for each tooth at the end of orthodontic treatment in both groups. Our findings showed a statistically significant difference between FAT1 and CAT1, at the expense of the second group, for the second premolar in the analysis of single occlusal contacts and in the evaluation of total occlusal contacts. Considering the antero-posterior ratio, higher values were reported in CAT for OCS, NOCS, half mm and one mm. Finally, when evaluating the asymmetries between the two hemiarches, no statistically significant differences were found between the two groups.

Previous studies compared conventional orthodontic and clear aligner therapy using different methods.

To evaluate pre- and post-treatment models and determine the outcomes of the Invisalign treatment, Kassas et al. [14] compared studies using ABO MGS. This paper showed a statistically significant difference in occlusal contacts between pre- and post-orthodontic aligner treatment groups with a *p*-value < 0.001.

In contrast, G. Djeu et al. [26] used the American Board of Orthodontics Phase III examination methodology, reporting 13 OGS points fewer in patients treated with aligners in comparison with those treated with braces, with lower occlusal contact scores.

ABO-OGS was the method chosen for occlusal contact comparison between CAT and FAT in several systematic reviews.

Zheng et al. [17] and Pithon et al. [18] conducted two meta-analyses of four articles each. They reported lower occlusal contacts in the CAT-treated group compared to the FAT-treated patients. In particular, the study by Zheng et al. pointed out that the Invisalign group was found to lose 13 OGS points more than the braces group, and FAT was reported to be less effective than CAT in achieving accurate occlusal contact.

Comparable results were found by Ke et al. [27], who conducted a systematic review. In this paper, a discrepancy between clear aligners and braces in treating malocclusion was described, with aligners being less effective than braces in producing adequate occlusal contacts.

In their meta-analysis, Papageorgiou et al. [19] compared fixed appliance and clear aligner therapy, considering studies that used two different analytic methods: ABO OGS and PAR index score. Considering ABO OGS, a meta-analysis of three studies showed a worsening in the category of occlusal contacts in the aligners group compared to the fixed group (MD: 3.1 points; 95% CI: 0.6–5.6 points; *p* = 0.02). Using the PAR index score, instead, no statistical differences were found between clear aligner and fixed appliance treatment at the end of therapy. As suggested by the authors, this discrepancy could be explained by the different components of the scores, with the PAR index assessing occlusion more vaguely than the ABO OGS [19]. The results from this pilot research study match the findings of Alhafi et al. [9]. These authors demonstrated that CAT and FAT treatments produced equivalent results for treating mild-to-moderate malocclusions. Our research results showed that total occlusal contacts at NOCS half-mm and one-mm thresholds maintained equal levels between the two treatment groups. The OCS values from the FAT group were higher, which indicates that fixed appliances reach interocclusal distances that are slightly better than those achieved by other appliances when the contact threshold reaches 50 μm or less. These small differences in surface area cannot be analyzed using the ABO score.

The research by Cohen-Lévy et al. [21] employed T-Scan digital occlusal analysis to show that CAT and FAT patients maintained equal occlusal contact quality throughout their treatment period from the beginning until six months of retention. Our results partially align with these findings, as no differences were observed in left–right asymmetry between groups. Our study found that CAT produced anterior–posterior ratios which were substantially higher than FAT. This outcome is different than that of Cohen-Lévy et al. They found that antero-posterior force distribution remains similar between the two groups. This may be due to different types of analysis. The 3D distance mapping system in our study measures the precise distance between opposing surfaces without considering force application.

A color map was used by Bowman et al. through Geomagic control X (3D Systems, Rock Hill, SC, USA) software to evaluate the occlusal contacts before and after orthodontic treatment with clear aligners, comparing the achieved occlusal contacts with the Clincheck prediction. When considering single-tooth contacts, no statistically significant differences were found for the central and lateral incisors, while a significant discrepancy was reported for canines, first premolars, second premolars, first molars and second molars. Moreover, significantly different posterior occlusal contacts and total occlusal contacts were observed between pre-treatment and post-treatment evaluations [22].

A major difference between predicted and achieved occlusal contacts was found by Bowman et al. in posterior teeth compared to anterior teeth. This result was supported by our study. Although we did not compare predicted and achieved occlusal values, we reported a major number of anterior occlusal contacts and a minor number of posterior occlusal contacts at the end of the therapy with clear aligners.

The research by Frenkel et al. [23] showed that FAT and CAT reduced tight and near contacts without creating any major differences between the two treatment groups. Our findings are broadly consistent, as no significant differences emerged at the NOCS, half-mm, and one-mm thresholds. Our study found that the FAT group showed substantially elevated OCS values when using a more precise measurement criterion of ≤50 μm, suggesting that fixed appliances may achieve closer interocclusal relationships detectable only at tighter distance criteria. Additionally, we observed significantly greater variability in post-treatment occlusal contacts in the CAT group for individual teeth, suggesting that aligner therapy may produce less predictable tooth-by-tooth outcomes compared to fixed appliances. This study has several limitations. The retrospective design and small sample size limit the generalizability of our findings. It must be acknowledged that occlusal contacts may undergo further refinement over time. The lack of long-term follow-up prevents assessment of occlusal settling. All these results must be confirmed in a larger study; the correct sample size for a confirmatory study would be 26 patients per group, according to our preliminary results. The variability observed in the present study will be useful for planning future research specifically powered to investigate temporal changes in occlusal contacts.

## 5. Conclusions

Within the limitations inherent to a pilot study, the present findings suggest the presence of potential differences between treatment modalities in the achievement of occlusal contacts, particularly for tight contacts and in the antero-posterior distribution. These results should not be interpreted as conclusive. This pilot study serves as a preliminary analysis for larger confirmatory studies and provides the effect size estimates necessary for appropriate sample size calculation.

## Figures and Tables

**Table 1:** Demographic and clinical characteristics of CAT and FAT.

Variable	CAT (Aligners)	FAT (Fixed Appliances)
Age	13.8 ± 0.6	13.2 ± 0.8
Sex		
Male	60%	70%
Female	40%	30%
Skeletal class		
Class I	50%	33.3%
Class II	50%	66.7%
Dental Class		
Class I	50%	33.3%
Class II	50%	66.7%
Vertical pattern		
Normodivergent	58.3%	50%
Hyperdivergent	33.3%	41.7%
Hypodivergent	8.3%	8.3%
Overjet (mm)	3.1 ± 0.8	3.1 ± 1.3
Overbite (mm)	3.1 ± 1.2	3.3 ± 1.2

**Table 2:** Single Tooth-MCP before and after treatment in CAT and FAT.

	AT0	AT1	FT0	FT1
Central incisor	0.53 ± 1.54	0.24 ± 1.17	0.92 ± 2.49	0.53 ± 0.34
Lateral incisor	1.34 ± 2.68	0.34 ± 0.92	1.33 ± 2.91	0.42 ± 0.33
Canine	1.37 ± 2.70	0.19 ± 0.79	0.28 ± 3.68	0.19 ± 0.15
First premolar	1.55 ± 3.00	0.18 ± 0.39	0.50 ± 3.15	0.18 ± 0.12
Second premolar	1.55 ± 2.98	0.19 ± 0.41	0.43 ± 3.31	0.07 ± 0.10
First molar	1.57 ± 2.86	0.16 ± 0.28	0.51 ± 3.05	0.09 ± 1.00

Measurements are in mm.

**Table 3:** Occlusal contacts for a single tooth after the orthodontic treatment with FAT and CAT.

Single Tooth	CAT T1 (Median ± IQR)	FAT T1 (Median ± IQR)	*p*-Value
Central incisors	0.24 ± 1.17	0.52 ± 0.33	0.55
Lateral incisors	0.34 ± 0.92	0.42 ± 0.33	0.78
Canine	0.19 ± 0.77	0.18 ± 0.15	0.79
First premolar	0.18 ± 0.38	0.18 ± 0.12	0.52
Second premolar	0.19 ± 0.41	0.06 ± 0.10	0.048 *
First molar	0.16 ± 0.28	0.09 ± 0.09	0.14

Measurements are in mm. A positive change indicates an increase in occlusal contacts. AT0 as aligner pre-treatment scan; AT1 as aligner post-treatment scan; FT0 as fixed pre-treatment scan; FT1 as post-treatment scan. The table shows median, interquartile range, and *p*-value. * Represents a statistically significant difference.

**Table 4:** Total occlusal contacts at T1.

Total Occlusal Contacts	CAT T1 (Median ± IQR)	FAT T1 (Median ± IQR)	*p*-Value
OCS	0.43 ± 0.84	1.27 ± 1.38	0.05 *
NOCS	34.30 ± 37.65	44.08 ± 36.65	0.14
Half mm	83.99 ± 62.05	89.78 ± 57.78	0.20
One mm	312.36 ± 143.60	364.64 ± 124.92	0.18

Measurements are in mm. The table shows median, interquartile range and *p*-value. * Represents a statistically significant difference. *p*-Value ≤ 0.05 was indicated as *.

**Table 5:** Non-parametric tests’ results.

Variable	Test	*p*-Value
OCS	Wilcoxon	0.05 *
OCS	Fligner-Killeen	0.03 *
NOCS	Wilcoxon	0.14
NOCS	Fligner-Killeen	0.70
Half mm	Wilcoxon	0.20
Half mm	Fligner-Killeen	0.72
One mm	Wilcoxon	0.18
One mm	Fligner-Killeen	0.23

The table shows results of the statistical tests performed on the sample. *p*-Value ≤ 0.05 was indicated as *.

**Table 6:** Antero-posterior ratio after treatment in CAT and FAT.

Antero-Posterior Ratio	CAT T1 (Median ± IQR)	FAT T1 (Median ± IQR)	*p*-Value
OCS	0.61 ± 1.56	0.00 ± 0.03	0.021 *
NOCS	0.35 ± 0.62	0.14 ± 0.13	0.012 *
Half mm	0.35 ± 0.42	0.16 ± 0.13	0.014 *
One mm	0.30 ± 0.17	0.20 ± 0.06	0.014 *

Measurements are in mm. The table shows median, interquartile range and *p*-value. * Represents a statistically significant difference. *p*-Value ≤ 0.05 was indicated as *.

**Table 7:** Asymmetries between left–right hemiarches.

Asymmetries Between Hemiarches	CAT T1 (Median ± IQR)	FAT T1 (Median ± IQR)	*p*-Value
OCS	1.00 ± 0.08	1.00 ± 0.68	0.13
NOCS	0.29 ± 0.48	0.21 ± 0.26	0.88
Half mm	0.22 ± 0.42	0.21 ± 0.27	0.47
One mm	0.12 ± 0.28	0.07 ± 0.02	0.23

Measurements are in mm. The table shows median, interquartile range and *p*-value.

## Data Availability

Data are unavailable due to privacy and ethical restrictions, but reasonable requests can be evaluated.

## Abbreviations

The following abbreviations are used in this manuscript:

CAT	Clear Aligner Treatment
FAT	Fixed Appliance Treatment
ABO	American Board of Orthodontics
MGS	Model Grading System
OGS	Objective Grading System
PAR	Peer Assessment Rating
OCS	Occlusal Contact Surface
NOCS	Near Occlusal Contact Surface
